# Protein Mutations and Stability, a Link with Disease: The Case Study of Frataxin

**DOI:** 10.3390/biomedicines10020425

**Published:** 2022-02-11

**Authors:** Rita Puglisi

**Affiliations:** UK Dementia Research Institute at the Wohl Institute of King’s College London, London SE59RT, UK; rita.puglisi@kcl.ac.uk

**Keywords:** Fe–S proteins, Fe–S cluster biogenesis, genetic diseases, missense mutations, protein stability

## Abstract

Protein mutations may lead to pathologies by causing protein misfunction or propensity to degradation. For this reason, several studies have been performed over the years to determine the capability of proteins to retain their native conformation under stress condition as well as factors to explain protein stabilization and the mechanisms behind unfolding. In this review, we explore the paradigmatic example of frataxin, an iron binding protein involved in Fe–S cluster biogenesis, and whose impairment causes a neurodegenerative disease called Friedreich’s Ataxia (FRDA). We summarize what is known about most common point mutations identified so far in heterozygous FRDA patients, their effects on frataxin structure and function and the consequences of its binding with partners.

## 1. Introduction

Several pathologies are linked to proteins with anomalous conformations. They can be distinguished into two categories: toxic gain-of-function and loss-of-function diseases. Neurodegenerative diseases characterized by metastable proteins prone to form toxic aggregates, i.e., soluble oligomers and fibrillar amyloid deposits, belong to the first group. The determination of protein stability may provide a measure of the propensity of the protein to aggregate. There are, however, several cases in which aggregation is not clearly linked to reduced stability. For instance, it has been shown that the ALS mutants of Cu, the Zn-superoxide dismutase apoprotein, do not all share reduced stability [1]. Additional factors must thus be identified and investigated to explain protein aggregation, for example, the lowering of the kinetic activation barrier for unfolding [2], internal dynamics and metal ion coordination [3,4], as well as environmental conditions, such as oxidative stress, pH shift and osmotic shock. In the second group of diseases, proteins are not functional or metastable and prone to degradation because of specific mutations. In this review, we examine this second group by taking the paradigmatic example of Friedreich’s ataxia (FRDA). Frataxin, the protein implicated in FRDA, has a role in the Fe–S cluster biogenesis and possesses a well-defined structure [5]. We aim to summarize what is known about this protein, to date. We will linger especially on its mutants and their correlation to FRDA. We propose a classification according to their effect on the folding, stability or function. We apologize in advance to the colleagues who have not been cited and suggest the reading of other recent reviews that complement the information reported here [5,6,7,8,9].

## 2. The Molecular Bases of Protein Stability

It can be generally assumed that proteins occur in two states: folded and unfolded. Protein stability is the capability of a protein to retain its native conformation under stress condition, such as the departure from room temperature, high pressure or the presence of denaturing chemicals. For all proteins, the transition between folded and unfolded species may occur both at temperatures higher and lower than room temperature and T_m_ and T_c_, the two temperatures at which there is the equilibrium between equal populations of the two species, are generally referred to as hot and cold unfolding temperatures, respectively [10]. The folded conformation is more populated in the temperature range between the two temperatures of melting, whereas unfolded forms predominate at temperatures higher than T_m_ or lower than T_c_. T_c_ is rarely determined since water usually freezes before this transition. On the other hand, the heat denaturation temperature, T_m_, is commonly taken as the best description of thermal stability, and a protein with a higher T_m_ is usually considered as more thermally stable. Strictly speaking, it corresponds to assuming a proportionality between ΔT_m_ and ΔΔG, which is true only if the corresponding stability curves at the two temperatures are parallel [11]. When this is not the case, the value of T_m_ describes only the thermal resistance. Proteins are generally active at temperatures up to their T_m_ and down to their T_c_, unless other effects take place, e.g., aggregation.

Over the years, a combination of different physical and chemical reasons has been identified to explain thermostability. A comprehensive description at the molecular scale of the mechanisms responsible for thermal and cold stability is extensively discussed in a review by Pucci and Rooman [12]. The hydrophobic effect constitutes the main driving force of protein folding and results from the tendency of hydrophobic amino acids to cluster together [13]. The two transitions are linked to the opposing changes in conformational entropy: the increase connected to the high temperature transition corresponds to a decrease in the low temperature transition. This difference has often puzzled many researchers, but it was made clear by Privalov [14] that the hydration of apolar side chains in the hydrophobic core compensates for this difference at a low temperature. In other words, the cold transition is mainly related to the weakening of the hydrophobic effect that becomes unfavorable below a certain temperature compared to hydrophobic–water interactions, whereas the hot denaturation is associated with an increment of conformational fluctuations [15]. Additionally, salt bridges contribute to improve heat resistance by lowering the de-solvation penalty at high temperatures [16,17], as well as the interactions between aromatic residues [18]. The formation of higher-order oligomeric structures is a possible strategy for protein thermal adaptation [19].

The knowledge of the difference in free energy between the folded and unfolded forms (ΔG) and the temperature of transition, T_m_, allow for a complete characterization of the thermal properties of a protein. However, the accurate determination of all thermodynamic parameters is only possible when both warm and cold denaturation are determined [20,21]. This information can be obtained by the observation of the folded fraction variation in the presence of a perturbing agent (e.g., temperature, pressure or denaturants). Monitoring can be performed using a variety of spectroscopic techniques. One of the most widely used techniques is circular dichroism (CD) spectroscopy in the 190–260 range, which reports changes in the content of secondary structures. Fluorescence spectroscopy is also often used to follow protein unfolding, usually exploiting the properties of the tryptophan residues. The side chains of these residues are specifically excited at 295 nm and their emission is sensitive to the chemical environment; it occurs at 350 nm when the residue experiences a polar and solvated environment, whereas it occurs at 320–340 in an apolar environment. The use of NMR spectroscopy to monitor unfolding has become progressively more important. Initially it was based on the properties of the so-called ring-current shifted peaks whose detection is strongly indicative in the folded species [22]. The measurement of the areas of these peaks in 1D NMR spectra is a sensitive way to measure folded populations, owing to the strict proportionality of peak area to concentration [23], whereas several limitations hampered the use of 2D NMR. Recently, Puglisi et al. [21] showed that 2D spectra can also be used reliably to monitor both cold and heat unfolding, and even to gather information from different parts of the protein [24].

Several studies exploiting the computational approaches have also been published to predict the temperature of thermal denaturation, T_m_. They are based on statistical analysis [25,26], molecular dynamics [27] or machine learning methods [28]. A new theoretical analysis was recently designed by Miotto et al. to predict thermal stability based on the structure only, and without any other a priori information [29].

Biologically active proteins, thought to be in a thermodynamically favorable conformation, are often marginally stable under physiological conditions [30]. Molecular chaperones assist proteins to efficiently fold by transiently shielding the hydrophobic amino acids belonging to the protein core in the native fold, but which are exposed in the non-native conformation. In the cell, the chaperones cooperate with proteostasis mechanisms that activate the degradation pathway for the misfolded protein. These defense mechanisms tend to decline during aging, facilitating the manifestation of misfolding diseases [31].

Understanding the mechanisms of protein folding is important to explain the extent to which protein thermal stability can be related to disease.

## 3. Friedreich’s Ataxia

Friedreich’s ataxia (FRDA) is an autosomal recessive hereditary ataxia that affects 1 individual out of every 50,000 [32]. The age at onset is usually before 25 years and the most common symptoms are the progressive ataxia of the four limbs, loss of tendon reflexes and position sense, pyramidal weakness of the legs, dysarthria, skeletal deformities (as scoliosis and pes cavus) and Babinski signs [33,34]. In addition, hypertrophic cardiomyopathy and diabetes or carbohydrate intolerance are observed in 30% of the cases [33,35,36]. However, the patients affected by FRDA present phenotypes that can range between different levels of severity and sometimes, if they do not meet all the essential criteria, they are classified as atypical. The disease has currently no treatment and the progression often leads to increasing disabilities and the eventual loss of independent ambulation, with most patients confined to a wheelchair by their late 20s. Death usually occurs before the age of 50.

The genetic alteration in FRDA was localized within chromosome 9q13-q21.1 [37], which encodes a protein called frataxin. This is a highly conserved protein found in all species, from prokaryotes to eukaryotes. It is localized in the inner mitochondrial membrane [38,39,40] and has a role in the cellular regulation of iron homeostasis.

A (GAA)_n_ triplet repeat expansion in the first intron of the frataxin gene, originally known as X25, is the most common mutation in FRDA [32], causing frataxin deficiency. In healthy individuals, the number of repeats is in the 6–36 range, whereas in FRDA it ranges from 70 to 1700 repeats, most commonly 600–900 [5]. A higher number of repeats directly correlates with an increase in disease severity, especially decreasing the age of disease onset [6,41]. At the cellular level, a general iron deficiency with an increase in mitochondrial iron import and accumulation [42,43], together with an increase in ROS [44] and disruption in both heme and Fe–S cluster production [45,46], are correlated to frataxin deficiency. The impairment of the frataxin function may damage mitochondria and also increase the chance of tumor formation. Frataxin missense mutations have indeed been identified in cancer tissues where tumor-initiating cells show a higher iron uptake [47].

## 4. Frataxin and Fe–S Cluster Biogenesis

Several diseases are associated with the disruption of cellular iron homeostasis, as both iron overload and deficiency are damaging to cells. The balance of iron in biological systems is thus of fundamental importance. Organisms evolved mechanisms to modulate free iron concentrations in the cell with the formation of prosthetic groups, such as heme and Fe–S clusters. Frataxin is supposed to play a role in the iron–sulfur cluster and heme biosynthesis [6]. FRDA patients show an Fe–S cluster deficiency from the beginning of the disorder, suggesting the direct role of frataxin in the Fe–S cluster assembly [46].

The Fe–S cluster prosthetic groups are of fundamental importance for protein structure, they play a role in electron transfer, substrate binding and activation, iron and sulfur storage, regulation of gene expression and sometimes in enzyme activity [48,49,50,51]. In bacteria, there are three separate pathways for cluster production: the nitrogen fixation machinery (Nif) [52,53], the Isc machinery for most cellular needs [54,55] and the Suf machinery, which contributes under stressed conditions [56]. The Isc system components have orthologs with high sequence homology in eukaryotes [50,57,58]. The main players are a desulfurase (Nfs1 in eukaryotes, IscS in bacteria) and a scaffold protein (Isu/IscU). The desulfurase is a symmetric dimer containing PLP. This enzyme converts cysteine into alanine, providing sulfur to the scaffold protein on which the cluster is assembled [59]. Two chaperones (HscA and HscB in bacteria), with which frataxin has an identical phylogenetic distribution [60], are supposed to help the releasing of the Fe–S cluster from the scaffold protein to the acceptor [61]. However, HscB has recently been shown to also interact with the desulfurase, opening a new question on its role [62]. Electrons necessary for sulfur liberation are provided by a ferredoxin (FdX in bacteria and Yah1 with the ferredoxin reductase Arh1 in eukaryotes) [57]. IscA/Isa is able to bind the nascent Fe–S cluster and it is supposed to be an alternative scaffold protein [63]. Recently, it has been shown that the complex made by the two homologs, IscA1/IscA2, interacting with IBA57 and in the presence of ferredoxin, assembles a [4Fe-4S]^2+^ cluster by the reductive coupling of two [2Fe-2S]^2+^ clusters [64,65]. Isd11 is a protein present only in eukaryotes. It plays a role as the adaptor between Nfs1 and the scaffold proteins, to promote sulfur release [66,67]. Another ancillary protein selectively present in prokaryotes is YfhJ [68]

Frataxin interacts with the proteins involved in the Fe–S assembly machinery [69,70,71,72] through a surface spreading from the acidic ridge to the β4-sheet, as identified by mutating some of the residues belonging to these regions (E108, E111, D124 and W155, N146) [73]. Initially, frataxin was supposed to contribute to the Fe–S cluster formation as an iron donor [74,75]. Independent evidence shows that frataxin could act as a regulator of the reaction speed with an effect that is iron-dependent, by tuning the quantity of clusters formed to match the apo-acceptor concentration [76]. Nonetheless, the role of frataxin is still controversial; although in vitro activity assays show that bacterial frataxin, named CyaY, inhibits the cluster formation in bacteria [77,78,79], frataxin activates the reaction in yeast and in the human protein system [80]. The interaction between frataxin, the desulfurase and the scaffold protein was revealed and extensively investigated [69,71,78]. In prokaryotes, YfhJ (also called IscX), along with frataxin, was suggested to be a regulator of the IscS activity depending on the iron concentration [81]. Intriguingly, in prokaryotes, CyaY competes with YfhJ and FdX for the same site on IscS, and the concentration of iron cations modulate the binding affinity to IscS [78,82,83].

## 5. Frataxin: Structure and Stability

**Figure 1:**
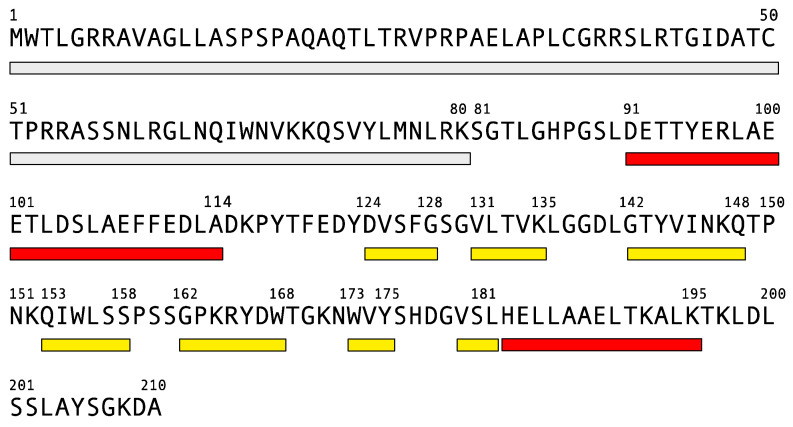
Human frataxin sequence and secondary structure, as reported for PDB 1EKG. Rectangles report the mitochondrial targeting signal and a spacer (gray), α-helices (red) and β-strands (yellow).

Human frataxin is a nuclear encoded protein of 210 amino acids. The sequence contains a 20-amino acid mitochondrial targeting signal and a spacer (Figure 1) that is removed from the mitochondrial matrix by a two-steps process, to produce mature frataxin [84].

**Figure 2:**
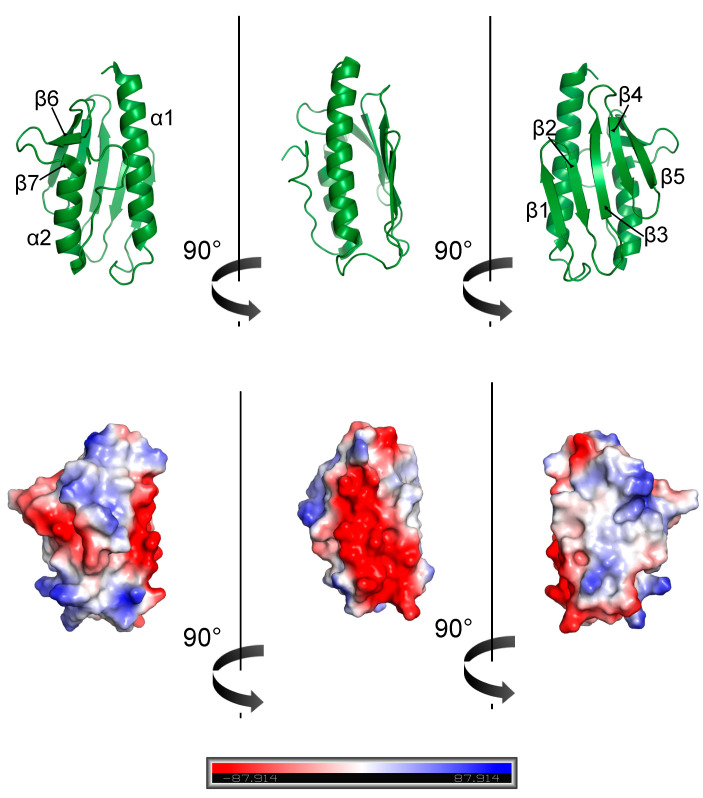
Human frataxin structure (PDB 1EKG) (top) and electrostatic surface (bottom).

Several solutions and crystal structures have been deposited in PDB for the bacterial and eukaryotic orthologues of frataxin [85,86,87,88,89,90,91,92]. The structures of yeast and human and bacterial frataxin orthologs are similar and consist of two regions: a compact globular C-terminal domain [89] and an intrinsically unfolded N-terminal tail (residues 81–92 in human frataxin). Overall, the domain presents a planar α−β sandwich structure motif (α1β1β2β3β4β5β6(β7)α2) with two terminal α-helices supported by a platform provided by five antiparallel β-strands and a sixth (and in human frataxin also a seventh) β-strand that intersects the planes (Figure 2, top).

Helices α1 and α2 are roughly parallel and secured by hydrophobic and aromatic residues (in human frataxin, L106, F110, and L113, and L186, L190 and L194, respectively). The spatial orientation is also stabilized by the interactions between hydrophobic amino acids on the β-sheet surface and additionally by the C-terminal tail (in human frataxin, T196, L198, and L200) [85,86,88,89,90]. As for human frataxin, the N-terminal tail of the yeast ortholog (Yfh1) is flexible and unfolded [6,93]. On the other hand, bacterial CyaY lacks any appreciable residues in the N-terminal, compared to α1 [7]. The three orthologues have a C-terminal tail with different lengths, without elements of a secondary structure [7,94] and with a higher mobility compared to the rest of the molecule, as confirmed by the smaller than average T_1_/T_2_ and small or negative NOE values [95], in agreement with larger rmsd of the solution bundle in these regions [89].

**Figure 3:**
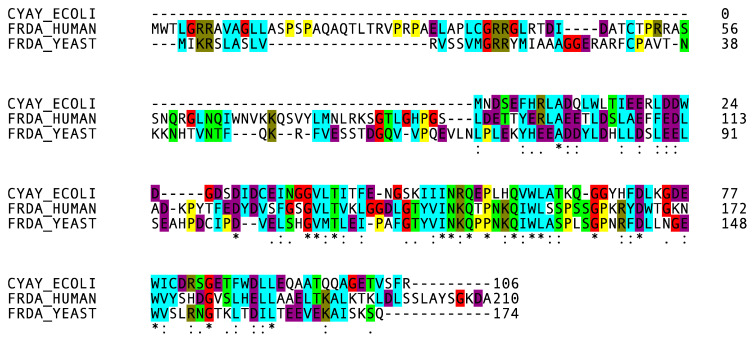
Multiple alignment of the three frataxin orthologues obtained with Clustal Omega and color-coded to emphasize the conserved sequence.

The strong structural similarity between the three frataxin orthologs corresponds to the high degree of conservation in the amino acid sequence [5] (Figure 3).

Mature human frataxin shares 36% of identical amino acids with yeast Yfh1 and 20% with bacterial CyaY. Two protein surfaces that are conserved throughout evolution were identified. An exposed surface, named as the “acidic ridge” and located in the α1 and β1 regions, is constituted by conserved negatively charged residues (E92, E96, E100, E101, D104, E108, E111, D112 and D115 in human frataxin), and is responsible for iron binding [89] (Figure 2, bottom). The other conserved surface forms an extended patch involving the positively charged and apolar residues from the beta-sheet [9]. The mutations that commonly result in FRDA are in these surfaces.

Despite the high degree of sequence homology and fold, the three frataxins have different stabilities in solution. While the bacterial and human orthologs have melting points at around 54 °C and 60 °C, respectively [94], yeast Yfh1 is less stable, with a melting point at around 35 °C and a temperature of cold denaturation above zero degrees (7 °C) [20,21,22]. Yfh1 is one of the few proteins whose cold denaturation is observable above water, freezing at nearly physiological conditions. The presence of salt and iron generally increases the stability, whereas the nature of the buffer has a minimal effect. Owing to the possibility of observing both unfolding transitions without the addition of denaturants, Yfh1 has extensively been used to investigate the mechanisms at the base of heat and cold denaturation [24,96,97,98]. The length of the C-terminal tail (longer in humans, intermediate in CyaY and shorter in Yfh1) influences the general stability of frataxin by insertion into the grove between α1 and α2 and providing additional contacts that help to stabilize the fold [16,94].

## 6. Frataxin Missense Mutation

The majority of the FRDA patients are compounds homozygous for (GAA)n expansion. Another small but significant number (2–8%) is observed to be heterozygous, with a (GAA)n expansion on one allele and a point mutation on the other [99,100]. To date, single base pair deletions, insertions and substitutions have been reported, counting at least 44 point mutations reported in the literature [101]. A list of the most common mutants is presented in Table 1.

**Figure 4:**
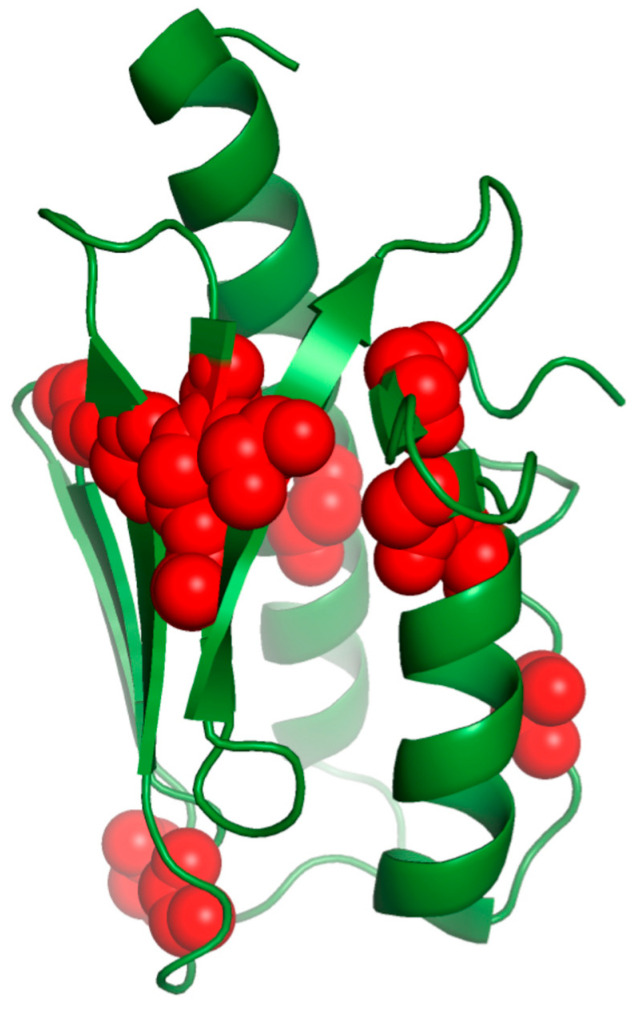
Frataxin residues substituted in FRDA mutants are colored in red.

FRDA patients homozygous for (GAA)n expansion usually present altered protein levels. The clinical presentation of the heterozygous patients can be either classical or with atypical or milder phenotypes, as missense mutations usually alter the features needed for the biological function or stability depending on the residues. The conserved amino acids of the hydrophobic core are usually essential for the folding; on the contrary, conserved exposed ones are usually involved in its function. In Table 2, we summarize the effects of the mutations on the frataxin structure and function and the disease phenotype in heterozygous patients carrying the most common point mutations identified to date in FRDA patients (Figure 4).

The mutations of the buried residues (L106, L182, H183 and L186) most likely disrupt the frataxin’s fold and cause FRDA disease.

This is the case for the replacement of a T with a C at position 317 in exon 3, which resulted in L106S missense. The patient carrying this mutation presented milder symptoms, such as the slow rate of disease progression, only lower limb weakness and no cardiac abnormalities or diabetes [103]. L106 belongs to the α1 helix and is conserved throughout the species. This is a nonconservative substitution of an apolar amino acid with a smaller polar residue with a lower tendency to form a helix [103]. The mutation from T to G was also reported. It causes the change from L106 to a stop codon (L106X) and leads to a truncated form of frataxin [102]. Patients carrying this mutation show a total deficiency of frataxin and typical FRDA with a severe course of the disease [32].

In other cases, individuals carry a C to T substitution altering the codon at position 182 and present a leucine replaced by the larger phenylalanine (L182F) in the α2 helix. They show atypical and mild symptoms, such as minimal dysarthria, little upper limb disfunction, absent ankle jerks, but reduced knee reflexes and progressively worsening lower limb ataxia [104]. Although L182 is supposed to be essential for the frataxin function, as it is a conserved residue, the L182F variant is still able to bind and activate the Fe–S machinery. On the other hand, the L182H mutation shows an alteration in the circular dichroism spectrum, suggesting a change in the secondary structure because of the substitution of a hydrophobic residue with a charged one.

H183 is a buried residue responsible for human frataxin stabilization, as it acts as a lock between the α1 and α2 helices, despite it is a non-conserved residue. Patients with H183R have been reported [100]. Although this is a conservative replacement, in which a positively charged amino acid is replaced by another one with similar characteristics, the steric difference between the two residues probably causes a strain disrupting the interaction.

Furthermore, the alteration in frataxin’s structure would arise from the mutation of β-sheet conserved residues with side chains directed towards the hydrophobic core, such as for L156 and W173.

L156 is highly conserved during evolution and a replacement by a proline (L156P) has been reported [100]. This is likely to profoundly affect the tridimensional structure, as proline has a high conformational rigidity and acts as a disruptor of the secondary structure. Consequently, the binding with IscU is affected [101]. W173 is also highly conserved and its replacement by a smaller residue, such as a glycine (W173G), may deeply affect protein folding [100] to the point that, when trying to produce it, the mutated protein is poorly expressed in the mature form [105].

On the other hand, the mutations of conserved exposed residues on the β-sheet plane (I154, N146, W155 and R165) may alter the ability of frataxin to bind its partners and disrupt the function.

Patients with an I154F mutation were found in a restricted area of southern Italy, probably because of the founder effect, in which the mutation is passed down from an individual to other generations. These patients present the typical FRDA phenotype [32,100]. The missense mutation affects a conserved residue located in β4, a highly conserved region involved in the interaction with Isd11 [105]. The maturation of this pathological variant is affected and results in the presence of insoluble intermediates [106]. The I154F mutant presents reduced stability with a T_m_ of 50.7 °C [95] and precipitates in the presence of iron [95,107].

Among the conserved residues on the β-sheet plane, N146, W155 and R165 are significative as they are adjacent in the structure, and the mutation of one has a consequence on the other. Interestingly, the patients carrying the N146K mutation presented classical features of FRDA; despite this, the frataxin mutant showed a stabilized native conformation with a T_m_ of 69.4 °C [9]. It has been suggested that the proximity of W155 and the replacing lysine may imbalance the electrostatic properties of the frataxin surface and affect the binding capacity of frataxin for its protein partners [73]. The W155R mutation causes a classical FRDA phenotype as well. W155 belongs to β4 and is an exposed residue responsible for the interaction with ISD11 that is disrupted after the mutation [105], as are the interactions with IscU and Nfs1 [73]. This mutant retains a native fold and has a slightly reduced stability [6,107] with a T_m_ of 61.4 °C [95]. The destabilization is expected from the deletion of a π–cation interaction between W155 and contiguous R165, and the electrostatic repulsion resulting from the insertion of arginine. On the contrary, patients with a compound heterozygous for the R165C substitution have a milder disease course (no dysarthria, gait disturbance and ankle jerks) [104]. This mutation occurs in a conserved region of β5. It is a nonconservative replacement, altering a basic amino acid to a hydrophobic non-charged one, which might also form an intermolecular disulfide bond and perturb the interaction with IscU [101].

As a result of their unique properties of backbone conformations, glycines are often positioned in loops and their substitution necessarily leads to the destabilization of the fold.

The G130V mutation is relatively common in FRDA [104]. People presenting frataxin with the G130V mutation have an atypical mild disease with an early onset, spastic gait, absence of dysarthria, retained tendon reflexes, mild or no ataxia and the slow progression of symptoms [100,104,108]. This suggests that, although the residue is highly conserved, the mutation only partially influences the frataxin function. G130V results in a large change in the T_m_ value (43.2 °C) [95] and it seems to affect the maturation of the protein [106]. G130 belongs to a turn between strands β1 and β2, and its backbone carbonyl oxygen forms a hydrogen bond with the amide of K147, which is involved in binding the scaffold protein IscU [101] and belongs to a ubiquitination site. The mutation of G130 thus results in a higher degradation of frataxin in the cell [109] and in the disruption of the interaction with IscU. In addition, the G130V mutation affects the frataxin iron affinity [95,107].

An FRDA patient with a compound heterozygous with an G137V substitution was also reported. It described an onset at 25 years of age and phenotypes, such as gait and trunk ataxia, mild dysarthria, absent tendon reflexes in the upper and lower limbs and impaired position and vibratory sense in the lower limbs [110]. Frataxin carrying the G137V mutation presented no effects on the structure or activity of the protein, but had a reduced conformational stability with a melting temperature of 46 °C. This is supposed to affect the efficiency of the folding process causing reduced levels of the active protein [110]. G137 is not an evolutionary conserved residue and is located in the C-terminal globular domain at the end of the β2 strand. Its mutation to a valine is supposed to have an important influence on the turn and, additionally, to cause steric hindrance with other residues. Interestingly, D122 packs against G137, suggesting that the two mutations have the same effect [110]. Similar to the patients carrying the G137V mutation, those found with the D122Y mutation had a mild and atypical disease [100]. D122 belongs to the N-terminus and its mutation does not affect the folding. However, by changing the polarity of the anionic surface, it reduces the protein stability [6] with a T_m_ of 50.4 °C [95]. The D122Y variant has a lower iron binding stoichiometry [95] and a perturbed interaction with the desulfurase [101].

As it was already described above, the C-terminal tail plays a crucial role in the stabilization of the fold [16,94]. A compound heterozygous patient was reported to present a base substitution that converted leucine to arginine at amino acid position 198 (L198R), located in an apolar environment in the C-terminus. The patient showed a typical FRDA phenotype [111]. L198 is a conserved residue and the mutation causes an alteration in charge with a consequent destabilization of the protein, with a T_m_ of 54.1 °C [112], due to the disruption of the interaction of the C-terminus with the α1 and α2 helices. L198R also showed a lower iron binding capability [112]. To determine the relevance of the frataxin C-terminus, other mutations at this position were also investigated (L198A and L198C). They all presented protein destabilization [113] as well as the absence of the C-terminus (residues 196–210) [94,112]. On the other hand, the truncation of frataxin at residue 193 determines FRDA with a rapid disease progression [114].

Such a variety of examples prove that similar dramatic or milder phenotypes do not necessarily derive from the same cause and, actually, may have a different origin at a molecular level. Depending on their localization in the structure, specific mutations can impair the function by disrupting the interaction with essential partners and decrease the protein stability leading to the degradation or disruption of the folding.

## 7. Conclusions

Thermal stability is of central importance in both science and medicine. The mechanisms by which protein heat resistance is modulated to allow the host organism to adapt to extreme environmental conditions is a topic of increasing interest in the research. Moreover, the investigation of protein stability has become more and more important, because protein misfolding and aggregation are key features of several neurodegenerative diseases, including Alzheimer’s disease, Huntington’s disease, Parkinson disease, cystic fibrosis and ALS [115]. In addition, several cancers are caused by the mutation and misfolding of proteins that are the key regulators of growth and differentiation [116].

In this study, we analyzed FRDA as an example of a disease that is caused by protein misfolding and a loss of protein function. Compound heterozygous patients with both point mutations and GAA expansion usually presented a severe FRDA phenotype, which resulted from reduced levels of functional frataxin. More attention to the mechanisms that lead to the misfunction of the frataxin gene, which affect frataxin maturation, folding, function or stability, will help in the future to establish a link between FRDA pathogenesis and phenotype, and inform treatment development.

## Figures and Tables

**Table 1:** List of deletions, insertions and substitutions in the coding sections of the frataxin gene responsible for the FRDA known, to date (more comprehensive tables can be found in Pook, 2000 [102] and Gellera, 2007 [99]).

Location	Nucleotide Change	Mutation	Effect
Exon 1	A→C at 1	1A→C	Incorrect initiation
	T→C at 2	2T→C	Incorrect initiation
	G→T at 3	3G→T	Incorrect initiation
	Deletion of C at 157	157delC	Frameshift
	Insertion of C after 157	157insC	Frameshift
Exon 2	GTCA→TTG at 202-205	202GTCA→TTG	Frameshift
Exon 3	T→G at 317	L106X	Nonsense
	T→C at 317	L106S	Missense
	Deletion of T at 317	317delT	Frameshift
	Deletion of 13bp between 340 and 352	340del13	Frameshift
	G→T at 364	D122Y	Missense
Exon 4	G→T at 389	G130V	Missense
	G→T at 410	G137V	Missense
	C→G at 438	N146K	Missense
	A→T at 460	I154F	Missense
	T→C at 464	W155R	Missense
	T→C at 467	L156P	Missense
Exon 5a	C→T at 493	R165C	Missense
	T→G at 517	W173G	Missense
	C→T at 544	L182F	Missense
	T→A at 545	L182H	Missense
	A→G at 548	H183R	Missense
	T→G at 593	L198R	Missense

**Table 2:** Summary of frataxin point mutations in FRDA: the location and effect on the protein structure, temperatures of unfolding (as a reference, T_m_ of the frataxin wild-type is 60 °C, as reported by Adinolfi, 2004 [94]), protein interaction site and FRDA phenotype.

Mutation	Location	Structural Effects	Other Effects	Tm (°C)	Affected Protein Interaction	Phenotype
L106S	α1	Steric strain due to replacement of a apolar residue with a smaller polar one.				milder symptoms
D122Y	loop	Change of a conserved negatively charged residue.	Lower iron binding stoichiometry.	50.4°C (Correia, 2008)	IscS	mild and atypical disease
G130V	turn *β*1–*β*2	Steric strain due to replacement of a glycine that is in a conformation not allowed to other residues.	Higher degradation of frataxin in the cell. Lower iron affinity.	43.2°C (Correia, 2008)		mild and atypical disease
G137V	turn *β*1–*β*2	Steric strain due to replacement of a glycine that is in a conformation not allowed to other residues and steric hindrance.	Lower efficiency of the folding process.	46°C (Faggianelli, 2015)		milder symptoms
N146K	*β*3	Electrostatic strain.		69.4°C (Castro, 2019)	IscU	classical
I154F	*β*4	Steric strain due to replacement of a hydrophobic residue by a larger one.	Maturation with increase of insoluble intermidiates.	50.7°C (Correia, 2008)	Isd11	classical
W155R	*β*4	Replacement of a bulky highly conserved aromatic residue with a positively charged one.		61.4°C (Correia, 2008)	Isd11/Nfs1 and IscU	classical
L156P	*β*4	Disruption in the *β*-sheet by introducing a proline.			IscU	classical
R165C	*β*5	Replacement of a conserved positively charged residue with a cysteine that is hydrophobic and might form intermolecular disulfide bond.			IscU	mild and atypical disease
W173G	*β*6	The introduction of a glycine affects the protein folding.	Poorly expressed.			classical
L182F	α2	Steric strain due to replacement of a hydrophobic residue by a larger one.	Prone to degradation.			mild and atypical disease
L182H	α2	Electrostatic strain due to replacement of a hydrophobic residue with a hydrophilic one.	Prone to degradation.			classical
H183R	α2	Strain due to replacement of a residue with a bulkier one.				classical
L198R	C-terminal region (CTR)	Electrostatic strain and disruption of the interaction of CTR with α1 and α2.	Lower iron binding affinity.	54.1°C (Faraj, 2014)		classical
